# Validation of a Real-Time PCR Assay for Fully Automated Detection of *Bacillus cereus* in Donor Human Milk

**DOI:** 10.3390/microorganisms13071640

**Published:** 2025-07-11

**Authors:** Gemma Aran, Vanessa Pleguezuelos, Margarita Blanco, Cristina Garcia, Mariama Jallow, Mar López, Sara Monge, Natalia Casamitjana, Eva Alonso-Nogués, Gloria Soria

**Affiliations:** 1 Banc de Sang i Teixits (Blood and Tissue Bank of Catalonia), 08005 Barcelona, Spain; vpleguezuelos@bst.cat (V.P.); crigarcia@bst.cat (C.G.); mjallow@bst.cat (M.J.); mmlopez@bst.cat (M.L.);; 2 Grup de Medicina Transfusional, Vall d’Hebron Institut de Recerca (VHIR), Vall d’Hebron Hospital Universitari, 08035 Barcelona, Spain

**Keywords:** BACARA^®^ plates, *Bacillus cereus*, cobas^®^ 6800 system, donor human milk, human milk bank, premature infants, real-time PCR

## Abstract

Donor human milk (DHM) can harbor microbial contaminants that cause serious infections in premature infants. *Bacillus cereus* is a pathogen frequently found in DHM, capable of forming spores that can resist Holder pasteurization (62.5 °C, 30 min). Since no microbial growth is acceptable in post-pasteurized DHM, microbiological testing of pre-pasteurized DHM provides information about its contamination level to determine if it should be accepted for pasteurization. Culture is the gold standard in microbiological control but it requires 24–48 h to provide results. In this study we developed and validated a non-commercial real-time PCR assay for the detection of *Bacillus cereus* (BC test) in DHM specimens on a fully automated high-throughput platform, the cobas^®^ 6800 system. The BC test showed excellent sensitivity and specificity, repeatability and linearity over an 8-log range and a low limit of detection in milk specimens, as well as good agreement with selective culture methods. BC test was then used to systematically control all milk donations (3439) over a 24-month period. *Bacillus cereus* was detected in 14.2% of DHM, with monthly rates ranging from 6 to 29% and a significantly higher incidence in warmer months. Incorporating this assay into our laboratory workflow improved efficiency and reduced turnaround time.

## 1. Introduction

Donor human milk (DHM) from a human milk bank (HMB) is the recommended feeding method for preterm infants when the mother’s own milk is not available, according to the World Health Organization, 2022 [1]. It provides essential nutrients and bioactive components that are critical for the growth and development of newborns [1,2]. However, DHM can sometimes be a vehicle for microbial contamination, which poses significant health risks to vulnerable populations, particularly premature infants. One such contaminant of concern is *Bacillus cereus* [3,4].

*Bacillus cereus* is a spore-forming, Gram-positive bacterium that is widely distributed in the environment. It produces enterotoxins that can lead to food poisoning. In healthy individuals, this may result in gastrointestinal symptoms such as vomiting and diarrhea [5,6]. *Bacillus cereus* is also associated with non-gastrointestinal pathologies such endophthalmitis, bacteremia, endocarditis or soft tissue and bone infections [7]. Premature infants have an increased susceptibility to serious infections due to their immature immune systems and underdeveloped gastrointestinal tracts [4,5]. In this vulnerable population, *Bacillus cereus* may cause life-threatening infections, including septicemia, meningitis, respiratory tract infection, hepatitis, endocarditis, endophthalmitis, encephalitis and necrotizing enterocolitis [4,8,9,10].

To mitigate the risk of microbial contamination, Holder pasteurization (HP) stands out as a widely adopted measure to ensure the microbiological safety of DHM. HP is the recommended treatment in international guidelines [11,12]. The HP process, involving heating milk to 62.5 ± 1°C for 30 min, effectively neutralizes numerous pathogens. Nonetheless, the spores of *Bacillus cereus* exhibit resistance to HP [3,8,9,10,11,12,13].

Since the establishment of the first HMB over a century ago, there has been a rapid growth in the number of countries where milk banking is practiced. There are currently 760 HMB in 70 countries [14]. However, there is a lack of global consensus regarding regulation of DHM. The three most common scenarios worldwide are an absence of any regulatory oversight, legislation of DHM as food or legislation of DHM as a substance of human origin [14]. As a consequence, key aspects in safety and quality such as donor selection, DHM handling and processing or microbial control differ among HMBs. In the absence of legislation, many HMBs operate according to guidelines issued by scientific associations, and thus apply different standards. For instance, the European Milk Bank Association (EMBA) recommends performing a pre-pasteurization and post-pasteurization microbial control [11] while the Human Milk Banking Association of North America only recommends a post-pasteurization microbial control [12]. In addition, as shown in a survey among HMBs in Europe [15], compliance rates with published guidelines is low and there is a wide variability in DHM processing and microbial screening.

Recent European legislation [16] has included DHM as a substance of human origin that has to abide by strict quality regulations provided by the European Directorate for the Quality of Medicines and HealthCare (EDQM) in technical guidelines such as the Guide to the quality and safety of tissues and cells for human application, 5th Edition, 2022 [17]. The EDQM guidelines emphasize the necessity of implementing standardized protocols for the collection, storage, and processing of DHM. These protocols include aseptic collection techniques, the use of sterile containers and strict adherence to hygiene practices by healthcare personnel and lactating mothers. Moreover, the guidelines advocate for regular microbiological screening of DHM donations. This involves systematic testing, pre- and post-pasteurization, to ensure the detection and quantification of potential microbial contaminants. The recommendations for pre-pasteurization involve taking a milk sample from each batch to test for microbiological bioburden. According to the European Milk Bank Association (EMBA), the milk should be accepted if it contains 10^5^ CFU/mL or less of non-pathogenic organisms and if no pathogens are detected [2]. Post-pasteurization, a sample of the milk should be taken for microbiological testing. No growth is acceptable in post-pasteurization microbiology cultures. There is a large variation in the microbiological screening practices of DHM among HMBs [14,15] but *Bacillus cereus* is the most frequent bacteria responsible for DHM being discarded [10,18,19,20,21,22].

Therefore, it is important to detect *Bacillus cereus* during regular pre-pasteurization microbiological testing, so that affected milk donations can be rejected at an early stage before pasteurization takes place in order to optimize resources. Milk donations are kept frozen and are thawed to be analyzed and pasteurized. Once they are thawed, a maximum conservation time of 24 h at refrigerated temperature is recommended [2]. Therefore, microbiological results are needed in less than 24 h. Although culture is the gold standard and microbiological controls of DHM are still based on traditional methods [22,23,24], since incubation is needed, turnaround time for results takes 24 to 48 h.

Molecular techniques such as real-time PCR (RT-PCR) have emerged as valuable tools for the rapid and sensitive detection of microbial contaminants in milk [25,26,27,28]. However, these methods are manual and time-consuming. In recent years, fully automated platforms with high-throughput capacity and ease of use have become available. One of such is cobas^®^ 6800 system, an automated system that performs nucleic acid extraction and RT-PCR and offers robust performance in detecting pathogens [29]. However, there is no commercial kit for the detection of *Bacillus cereus* in automated platforms.

With the aim of improving the efficiency and turnaround time of testing and thus the safety of DHM for vulnerable infants, we implemented and validated a RT-PCR for the automated detection of *Bacillus cereus* in donor human milk on the cobas^®^ 6800 system (BC test). The validation design was prepared following the guidelines published by the European Medicines Agency, specifically ICH Q14 Analytical procedure development [30] and ICH Q2 Validation of Analytical Procedures [31]. The following parameters were evaluated: sensitivity and specificity, cross-contamination, repeatability, linearity and limit of detection in milk specimens. Furthermore, we compared its performance with that of selective culture on BACARA^®^ plates. After this initial validation, we tested the routine performance by prospectively analyzing all DHM samples over a 24-month period.

## 2. Materials and Methods

### 2.1. Donor Human Milk Samples 

#### 2.1.1. Donor Screening Criteria and Donor Human Milk Processing

The DHM samples used in this study were obtained from January 2022 until December 2024 by the HMB of the Blood and Tissue Bank (BST), in Barcelona, which is responsible for the collection, processing, storage and distribution of DHM in Catalonia. Mothers who donated had been accepted as milk donors based on their clinical and social history, as well as the results of the serological tests required for donation.

The donors collected DHM using a breast pump and 150 mL sterile polypropylene containers (Medela S.L., Madrid, Spain) provided by the HMB. DHM was stored in food-grade polypropylene containers with screw caps and frozen in the donors’ domestic freezers at temperatures below –18 °C for up to 4 weeks. The HMB subsequently collected the donations using certified biological transport, with validated containers, ice packs, and dry ice, thereby ensuring the cold chain during DHM transportation. Once received at the HMB, DHM was stored at –20 °C. Within 3 months from the date of expression, DHM was selected and thawed in a cold chamber at 4 °C for 24 h. Aliquots for analysis were prepared under aseptic conditions inside a Grade A laminar flow hood (NuAire, Inc., Plymouth, MN, USA) and transferred into sterile 5 mL tubes. DHM aliquots intended for pasteurization were packaged in 50, 100 and 250 mL polypropylene containers (Sterifeed^®^, Totnes, UK) and sealed with a thermal sealing machine (ICS600, Sterifeed^®^, Totnes, UK). The samples were then subjected to a temperature of 62.5 °C for 30 min following the Holder pasteurization method using a pasteurization unit (S90, Sterifeed^®^, Totnes, UK). After pasteurization, new aliquots were prepared for analysis following the same procedure.

#### 2.1.2. Samples and Controls Preparation 

The DHM specimens used in this study were leftover samples from daily routine. Samples were stored at −80 °C until measured.

Negative post-pasteurization DHM samples were pooled, anonymized, aliquoted, and frozen at −80 °C until needed as negative controls. Positive controls were prepared by spiking negative controls with *Bacillus cereus* NCTC 7464 (BioMérieux, Craponne, France) to produce high-level or low-level positive samples, as required.

### 2.2. Microorganisms

The microbial strains used in this study were either standard commercial strains obtained from BioMérieux or microorganisms grown from DHM samples during microbial testing at BST and identified by Matrix-Assisted Laser Desorption Ionization Time of Flight (MALDI-TOF) using the MaldiBiotyper Sirius (Brucker, Bremen, Germany). Strains were kept frozen with 20% glycerol at −80 °C until needed.

### 2.3. RT-PCR Detection of Bacillus cereus

#### 2.3.1. Primers and Probes

The presence of *Bacillus cereus* group bacteria in DHM samples was determined through the amplification and detection of specific DNA by RT-PCR with fluorescent probes, using a modification of the method described by J.F. Martínez-Blanch et al. [25]. Oligonucleotides were synthesized by Life Technologies (Thermo-Fisher Scientific, Waltham, MA, USA).

#### 2.3.2. Manual Procedure

Total DNA was purified from an aliquot of DHM using a standardized extraction protocol. Initially, 1 mL of milk was centrifuged for 10 min at 8000 rpm (approximately 5000× *g*). After centrifugation, 0.75 mL of the supernatant was discarded, leaving approximately 250 µL of concentrated milk. The pellet was resuspended using a vortex to ensure thorough mixing. DNA extraction was performed with a QIAamp DNA Mini Kit (Qiagen, Hilden, Germany), following manufacturer’s instructions. Following DNA isolation, amplification was performed using sequence-specific primers (FW-5′-GGATTCATGGAGCGGCAGTA-3′, RV-5′-GCTTACCTGTCATTGGTGTAACTTCA-3′) and a probe (5′-FAM-CGAAACAAGATTACICT-3′) targeting the *Bacillus cereus plc* (phosphatidylcholine-specific phospholipase C) gene sequence [25]. The internal control TaqMan^®^ RNase P Detection Reagents Kit, 20× (Thermo-Fisher Scientific) was added to ensure an accurate amplification. To perform the RT-PCR, the TaqMan^®^ Universal PCR Master Mix (Applied Biosystems, Thermo-Fisher Scientific) was used following the manufacturer’s instruction. Amplification conditions were as follows: 120 seconds (s) at 50 °C, 10 min at 95 °C, 50 cycles of 15 s at 95 °C and 60 s at 60 °C and a final cooling step of 10 s at 40 °C. The amplification process was monitored on channel FAM and HEX by measuring the increase in fluorescence with the LightCycler^®^ 480 system (Roche, Basel, Switzerland), allowing the detection and quantification of the target DNA sequence.

#### 2.3.3. Automated Procedure

A cobas^®^ 6800 system was used to automate the extraction and RT-PCR process. A test for the automated detection of *Bacillus cereus* in human milk was developed using the same primers and probe as in the manual procedure described above (BC test). The development involved preliminary testing of cobas^®^ 6800 system reagents and RT-PCR conditions using the LightCycler^®^ 480 system. Verification of primer–probe compatibility was carried out by testing the cobas^®^ Omni Optimization Kit (Roche) in conjunction with the primers and probes specific for *Bacillus cereus*. A utility channel analysis package (UCAP) was created and stored using the cobas^®^ Omni Utility Channel Optimization tool v4.1 (Roche). The UCAP contains the default PCR profile, the selected sample type, a defined input volume and the relative fluorescence intensity (RFI) cut-off value for use on the cobas 6800 system. The Utility Channel Master Mix Reagent (Roche) used includes sequence-specific primers and a probe for the amplification of the Internal Control (IC). The IC is spiked in automatically during extraction, thus acting as a built-in full-process control for each reaction. In this system, the signal for *Bacillus cereus* is detected using the FAM fluorochrome (channel 2), and the IC is detected with Cy 5.5 (channel 5). Results were generated automatically by the system using the settings defined by the UCAP. The primers and probe were loaded in the cassette of the kit cobas 58/68/88 utility channel 192t IVD (Roche) at a final concentration of 0.3 µM, and 0.2 µM, respectively.

Several cobas^®^ 6800 system extraction parameters were evaluated to determine the most suitable one. After optimization, the final procedure involved the following steps. First, centrifuge 1 mL of DHM for 10 min at 8000 rpm (approximately 5000× *g*). Next, discard 0.6 mL of the supernatant, leaving approximately 0.4 mL. Then, resuspend the pellet in the remaining milk and transfer it to a tube with a “U” bottom that is compatible with the cobas^®^ 6800 system. These tubes were labeled with a barcode so that the cobas^®^ 6800 system could read the sample number. Amplification conditions were as follows: a pre-PCR incubation at 120 s at 55 °C, 360 s at 60 °C and 240 s at 65 °C, followed by 5 amplification cycles of 5 s at 95 °C and 30 s at 55 °C and 45 cycles of 5 s at 91 °C and 25 s at 60 °C. This quick and automated method yields results within 2.5 h.

### 2.4. BC Test Performance Assesment

After conducting a risk assessment to identify the parameters that could impact the relevant performance characteristics of the assay, a validation study was designed according to established guidelines [30,31].

The sensitivity or inclusivity and specificity or selectivity was evaluated through absence of interference with milk matrix as well as comparison of BC test results to microbiological culture results, which is the reference procedure. The acceptance criteria was complete agreement between both methods. Two different studies were performed: first using collection strains and then using real DHM samples.

Negative post-pasteurization DHM, proven by culture to harbor no microbes, was pooled, anonymized, aliquoted and kept frozen at −80 °C until needed. After thawing overnight at 4–8 °C, aliquots were spiked with 10^4^ concentrations of *Bacillus cereus* (NCTC 7464), *Staphylococcus aureus* (ATCC 6538), *Pseudomonas aeruginosa* (ATCC 9027), *Candida albicans* (ATCC 10231), *Bacillus spizizenii* (ATCC 56024), *Aspergillus brasiliensis* (ATCC 16404) and *Escherichia coli* (ATCC 8739) (BioMérieux) to study specificity and sensitivity.

DHM leftover samples from the daily routine, microbiologically characterized by culture, were also used to study sensitivity and specificity. To test sensitivity (inclusivity) the panel included 20 samples that were positive for the following *Bacillus cereus* group species: *Bacillus cereus* and *Bacillus mycoides.* To test specificity (cross-reaction), the panel included 20 samples positive for microorganisms frequently found in DHM—*Staphylococcus* sp., *Acinetobacter* sp., *Klebsiella* sp., *Serratia* sp., *Enterobacter* sp., *Pseudomonas* sp.—and negative for *Bacillus cereus* group. The microbiological composition of these samples is shown in Supplementary Table S1.

The precision studies included repeatability and intermediate precision. Repeatability (inter-run variability) was assessed by analyzing a high positive (10^6^ CFU/mL of *Bacillus cereus*) milk control and a low positive (10^3^ CFU/mL) milk control in triplicate. Intermediate precision (inter-run variability) was tested by analyzing a negative milk control and a positive milk control (10^4^ CFU/mL of *Bacillus cereus*) in 15 different runs over a month and conducted by different analysts. Although there are no specific standards, we established a maximum of 5% variation in Cts as acceptance criteria in the validation protocol, based on published coefficients of variation for commercial PCR tests [32].

To evaluate precision, positive and negative controls were prepared as described in Section 2.1. Each control was then aliquoted and kept frozen until used.

Accuracy was evaluated across the reportable range of the assay through comparison of the BC test results with microbiological culture results, both in saline solution and in milk matrix, with a spiking study. A linear relationship between *Bacillus cereus* concentration and BC test results in the studied range was assessed and should demonstrate the analytical procedure capability to obtain values that are proportional to the true sample values. Test results were evaluated by calculation of a regression line by the method of least squares. A plot of the data, the correlation coefficient, y-intercept and slope of the regression line, as well as PCR efficiency, were calculated. The acceptance criteria were defined as linearity over the range 10^6^ to 10^0^ CFU/mL [33], with an R^2^ > 0.98 and PCR efficiency of 80 to 120% [31].

To study linearity, correlation with bacterial concentration and limit of detection, a tube of thioglycollate medium (BioMérieux) was inoculated with *Bacillus cereus* NCTC 7464 (BioMérieux) and incubated for 18 h. The following day, 10-fold serial dilutions of the culture were prepared in saline solution 0.9% (Fresenius Kabi, Bad Homburg, Germany), up to a dilution factor of 10−8. Each dilution was then subjected to BC test in duplicate using the cobas^®^ 6800 system and LightCycler^®^ 480 system, and parallel cultures were plated to determine the concentration of viable bacteria.

To study linearity, correlation with bacterial concentration and limit of detection in a milk matrix, a concentrated positive DHM control was prepared by spiking negative DHM controls with *Bacillus cereus* NCTC 7464 (BioMérieux) at a concentration of 10^6^. This positive control was serially diluted with negative DHM to obtain concentrations ranging from 10^6^ to 10^0^ CFU/mL. Each dilution was then tested in triplicate by RT-PCR analysis using the cobas^®^ 6800 system, and parallel cultures were plated to verify the concentration of viable bacteria. To estimate the limit of detection, quantified milk samples were serially diluted with negative milk to obtain final concentrations ranging from 20 CFU/mL down to 0.01 CFU/mL. Since 1 mL is the volume used in the test, these concentrations are equivalent to 20–0.01 copies per reaction. Each dilution was tested in triplicate, except the concentration of 1 CFU/mL that was tested 10 times.

Robustness, which is a measure of the method’s capacity to meet the expected performance criteria during normal use, was assessed during precision studies. It was furthermore complemented with cross-contamination studies to evaluate if highly positive samples influenced the results of negative samples. To that effect, 2 negative DHM controls were placed next to 2 positive DHM controls (10^4^ CFU/mL of *Bacillus cereus*) and were tested in 15 independent experiments.

### 2.5. Pre-Pasteurization Milk Culture

BST adheres to the recommendations outlined in the Guide for the Quality and Safety of Tissues and Cells for Human Application [17], and the guidelines provided by the European Milk Bank Association (EMBA) [2,11]. These recommendations emphasize the importance of microbiological control both before HP. The primary objective of pre-pasteurization milk culture is to quantify and characterize the microbial load (bioburden), serving as the basis for determining whether the milk should proceed to processing. The criteria for acceptance of pre-pasteurization milk in BST are as follows:

- Total aerobic microorganisms: ≤1 × 10^5^ colony-forming units (CFU) per milliliter (mL);
- Staphylococcus aureus: ≤1 × 10^4^ CFU/mL;
- Enterobacteriaceae: ≤1 × 10^4^ CFU/mL;
- Bacillus cereus: <1 CFU/mL.

The microbiological control of pre-pasteurized milk is carried out using the plate count, surface-spread method, employing the following culture media: Trypticase Soya Agar (TSA) for total microorganisms, MacConkey Agar (MCK) for *Enterobacteriaceae*, Mannitol Salt Agar, also known as Chapman Agar (CHA), for *Staphylococcus aureus* and BACARA^®^ for *Bacillus cereus*. The plates used were from BioMérieux.

Within a laminar flow cabinet and utilizing an aseptic technique, DHM samples were diluted in a saline solution of 0.9% at a maximum dilution of 1:100 using a dilution bank and ensuring homogenization at each step. For inoculation, 1 mL of undiluted milk was plated on BACARA^®^ plates, and 0.1 mL of the 10^−2^ dilution was plated on CHA, MCK and TSA plates. The plates were then incubated at 36 °C ± 1 °C for 24 h. The colonies were counted and calculations were made with Modulab Information System (WERFEN, Barcelona, Spain) to obtain the total microbial count and the count of *Enterobacteriaceae*, *Staphylococcus aureus* and *Bacillus cereus*. When needed, colony identification was performed by MALDI-TOF using the MaldiBiotyper Sirius (Brucker).

### 2.6. Quality System

The HMB and the microbiology laboratory at BST comply with Good Manufacturing Practices and Good Laboratory Practices. Therefore, there is a quality system in place; all equipment is qualified before being used and regular calibration and maintenance is performed, the personnel is trained and competency is assessed, the analytical methods are validated before using them with appropriate validation protocols, there are written standard operating protocols for all procedures, all data, records and reports are archived to ensure traceability and data integrity. Internal and external audits periodically verify the quality system.

To avoid contamination, all operations are performed in a flow cabinet with aseptic technique. Positive and negative controls are included daily in all procedures: culture-based and molecular. Furthermore, we participate in external control programs for microbiological testing.

### 2.7. Data Analysis

All data were automatically analyzed by the cobas^®^ 6800 system (software version 1.4.7.1003) using the criteria defined in the UC analysis package. PCR efficiency was determined with the formula E = −1 + 10^(−1/slope)^. Graphs and statistical analysis were performed using GraphPad Prism version 5 (GraphPad Software, San Diego, CA, USA).

## 3. Results

### 3.1. Optimization of BC Test in cobas^®^ 6800 System

Implementation and optimization of the BC test in the cobas^®^ 6800 system is described in the methods Section 2.3.3. Good correlation between manual DNA extraction and RT-PCR with another instrument (LightCycler 480) and automated extraction and RT-PCR with cobas^®^ 6800 system was demonstrated, with an (R^2^) value of 0.99..

Since milk is a complex matrix prone to PCR inhibition, several pre-treatment and cobas^®^ 6800 system extraction parameters were evaluated to determine the most suitable one. The different conditions tested were as follows:

1. Raw milk sample (1.5 mL), extraction volume 850 µL;
2. Centrifuge 1 mL of milk and remove 450 µL, leaving 550 µL for extraction volume of 350 µL;
3. Centrifuge 1 mL of milk and remove 600 µL, leaving 400 µL, which is supplemented with 150 µL of sterile water to account for dead volume, for an extraction volume of 350 µL;
4. Centrifuge 1 mL of milk and remove 600 µL, leaving 400 µL for an extraction volume of 150 µL;
5. Centrifuge 1 mL of milk and remove 750 µL, leaving 250 µL, which is supplemented with 150 µL of sterile water to account for dead volume, for an extraction volume of 150 µL.

The results obtained are summarized in Table 1. In all cases, the tested samples were the same positive and negative controls of *Bacillus cereus* in donor human milk. Since the system has a dead volume of 300 µL, the total sample volume prepared must account for this dead volume to ensure there is enough sample for the procedure.

Based on these results, the optimal extraction volume was determined to be 150 µL. Conditions 1 and 2 were deemed not adequate because the internal control amplification failed, which indicates a PCR inhibition. This is due to the interference of the donor human milk matrix with DNA extraction and/or the detection of *Bacillus cereus* by RT-PCR. The data suggest that the best method to analyze this type of sample is to centrifuge it to remove as much of the fatty milk matrix as possible. Condition 3 allowed internal control amplification but showed a significantly higher Ct (31.98 ± 0.45) for *Bacillus cereus* than either conditions 4 (28.48 ± 0.16) (*p* = 0.0058) or 5 (27.45 ± 0.51) (*p* = 0.0015). On the other hand, there was no difference (*p* = 0.1135) between conditions 4 and 5. Therefore, to minimizing the need for excessive handling, case 4 was elected, since no supplementation was required. All subsequent experiments were conducted using condition 4.

### 3.2. Analytical Sensitivity and Specificity

Initial evaluation of sensitivity and specificity of BC test involved testing a pure culture (10^4^ CFU/mL) of known commercial strains by duplicate. *Bacillus cereus* (NCTC 7464) gave positive results, with a mean Ct of 25.57 ± 0.15 (CI: 25.19–25.95), while *Staphylococcus aureus* (ATCC 6538), *Pseudomonas aeruginosa* (ATCC 9027), *Candida albicans* (ATCC 10231), *Bacillus spizizenii* (ATCC 56024), *Aspergillus brasiliensis* (ATCC 16404) and *Escherichia coli* (ATCC 8739) yielded negative results (no amplification, Ct > 45).

The BC test was then tested against a selection of 20 DHM samples that had been determined by culture to contain species of the *Bacillus cereus* group at concentrations ranging from 10 to 11,000 CFU/mL. All samples yielded positive results. Specificity was tested against a selection of 20 DHM samples that had been characterized by culture and had a total load of 10^5^–10^6^ CFU/mL of microorganisms but were free of *Bacillus cereus*. No cross-reactivity was detected, even in the presence of high levels of other microorganisms that are generally found in milk, such as *Staphylococcus*, *Micrococcus*, *Acinetobacter*, *Klebsiella*, *Serratia*, *Enterobacter*, *Pseudomonas*, *Stenotrophomonas,* etc. All samples yielded negative results (no amplification, Ct > 45). The results are shown in Supplementary Table S1.

The acceptance criterion, which was complete agreement between both methods, was fulfilled in both experiments.

### 3.3. Cross-Contamination

As a test for robustness of the analytical method, cross-contamination was evaluated. The experiment assessed if the cobas^®^ 6800 system would yield false positive results for negative samples due to carry over between physically close samples. In 15 independent experiments, negative DHM controls were placed next to positive DHM controls in the entrance rack. Negative controls yielded negative results and therefore no cross-contamination was detected.

### 3.4. Precision

Analytical precision was evaluated using the cobas^®^ 6800 system to determine the consistency of results. Intra-run variability was assayed by analyzing a high positive (10^6^ CFU/mL of *Bacillus cereus*) milk control and a low positive (10^3^ CFU/mL) milk control in triplicate. The mean Ct for the high positive control was 18.93 ± 0.29 (standard deviation), resulting in a coefficient of variation (CV) of 1.53%. The mean Ct for the low positive control was 29.45 ± 0.25, with a CV of 0.85%. Results are shown in Table 2.

Inter-run variability (intermediate precision) was tested by analyzing a negative milk control a positive milk control (10^4^ CFU/mL) in 15 different runs over a month. The cobas^®^ 6800 system produced identical qualitative results in every instance. Amplification curves showed highly reproducible results with minimal day-to-day variation. Additionally, the internal control for all tests met the required standards. The mean Ct for the positive control was 26.58 ± 0.61, with a CV of 2.31%. Results are shown in Table 2. Overall, these metrics indicate low variability and therefore high reproducibility. Analytical precision was satisfactory, with standard deviations < 1 Ct and variation coefficients < 3% for positive milk samples over a month. Coefficients of variation were below 5% and therefore fulfilled the acceptance criteria.

### 3.5. Linearity, Limit of Detection and Correlation with Bacterial Concentration

**Figure 1:**
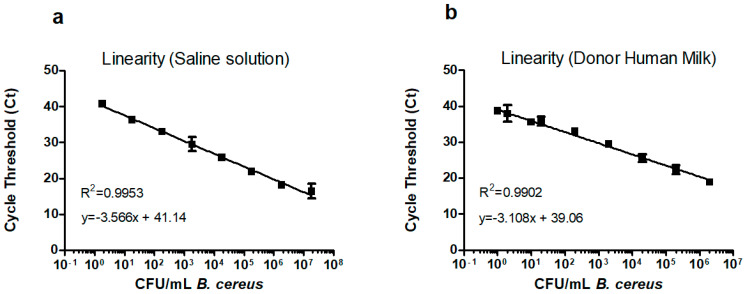
Linearity. Plotted are the linear regression lines and cycle threshold (Ct) mean values for each *Bacillus cereus* concentration expressed in colony-forming units (CFU/mL). Data from different experiments is shown together. Error bars show 95% confidence intervals. (**a**) Saline solution. (**b**) Donor human milk.

To further validate the DNA extraction and RT-PCR technique utilized by the cobas^®^ 6800 system, we conducted an experiment to determine the correlation between RT-PCR results and viable bacterial concentration as assessed by traditional culture methods. This experiment also aimed to evaluate the linearity and detection limit of the RT-PCR assay. The results are shown in Figure 1. A *Bacillus cereus* NCTC 7464 overnight culture was serially diluted 10-fold with saline solution to obtain 8 dilutions. Each dilution was tested in duplicate on a cobas 6800 system. The initial culture concentration of *Bacillus cereus* was determined to be 1.8 × 10^7^ CFU/mL by plating on TSA, as described in the methods Section 2.4. The BC test yielded positive results for all dilutions except for 10^−8^, indicating the system’s ability to detect bacterial concentrations as low as 1.8 CFU/mL. At the 10^−8^ dilution, which corresponds to 0.18 CFU/mL (<1 CFU/mL), no amplification was detected (Ct > 45). All coefficients of variation were <3.5%. The assay was linear over a range of 8 log units from 10^7^ to 10^0^ CFU/mL. The correlation between Ct values in cobas^®^ 6800 system and bacterial concentration was robust, with an R-squared (R^2^) value of 0.99, indicating a very strong linear relationship (Figure 1). The PCR efficiency was 90.73%.

To verify the linearity and limit of detection in milk specimens, a spiking experiment was performed with *Bacillus cereus* NCTC 7464. A concentrated positive DHM control was prepared at a concentration of 2 × 10^6^ and it was serially diluted 10-fold with negative DHM to obtain concentrations ranging from 2 × 10^6^ to 0.02 CFU/mL. The concentration was verified by plating on TSA. Triplicates of each dilution were tested on a cobas^®^ 6800 system. The BC test yielded positive results for all replicates from 2 × 10^6^ to 2 CFU/mL. All coefficients of variation were <2.5%. Only one out of three replicates at a concentration of 0.2 CFU/mL was positive. No replicates were positive at 0.02 CFU/mL.

To further assess the limit of detection (LOD) and limit of quantitation (LOQ), another spiking experiment was performed. A suspension of *Bacillus cereus* NCTC 7464 in DHM was prepared at concentration of 1 CFU/mL. Ten replicates were measured. The target DNA was detected in 10 of 10 measurements. The coefficient of variation was 0.4%. These results confirmed that the RT-PCR assay in a cobas^®^ 6800 system can detect *Bacillus cereus* at a concentration of 1 CFU/mL.

The assay was linear in milk over a dynamic range of 7 log units from 2 × 10^6^ to 1 CFU/mL. The limit of detection and quantification was confirmed to be 1 CFU/mL. The correlation between Ct values in cobas^®^ 6800 system and bacterial concentration was robust, with an (R^2^) value of 0.99. The PCR efficiency was 109.77% for milk samples. The results are shown in Figure 1.

The acceptance criteria for linearity (R^2^ > 0.98, PCR efficiency 80 to 120%) were fulfilled both in saline solution and milk.

### 3.6. Comparative Analysis of BC Test and BACARA^®^ Plate to Detect Bacillus cereus in Donor Human Milk

A comparative analysis of two methods for detection of *Bacillus cereus* in DHM was performed: BC test and BACARA^®^ plate. The cobas^®^ 6800 system is a highly automated, high-throughput instrument that uses RT-PCR technology to detect the presence of specific bacterial DNA. In contrast, the BACARA^®^ method is a traditional microbiological technique that relies on chromogenic selective culture media to isolate and identify *Bacillus cereus* colonies based on their morphological characteristics and is considered the gold standard. The comparison was performed from October 2023 until December 2024 by analyzing a total of 2434 pre-pasteurization milk samples with both methods. The results are shown in Table 3.

The sensitivity of the RT-PCR method was found to be 0.87 (95% CI: 0.83–0.91). Specificity was 0.94 (95% CI: 0.92–0.95). The positive predictive value (PPV) was determined to be 0.66 (95% CI: 0.62–0.71). The negative predictive value (NPV) was 0.98 (95% CI: 0.97–0.99), signifying the probability that a negative RT-PCR result accurately indicates the absence of *Bacillus cereus* by culture. Out of the 2434 samples, 2257 (93%) yielded concordant results between the two systems, resulting in a Cohen’s Kappa coefficient of 0.71 (95% CI: 0.67–0.75) that demonstrates substantial agreement [34] between the RT-PCR method and the BACARA^®^ culture plate. Due to insufficient sample volume, it was not possible to repeat the testing of discrepant samples. However, further analysis of discrepant results revealed that 35 out of 39 (89.7%) of RT-PCR negative results had a low colony count in BACARA^®^ culture of less than 10 CFU/mL. On the other hand, 99 out of 138 (71.7%) of BACARA^®^ negative results had a high Ct (>40) in RT-PCR, that corresponds to less than 1 CFU/mL. It is inferred that discrepant samples had a very low concentration of *Bacillus cereus*. This low concentration likely accounts for the non-repeatability of the results, irrespective of the method of analysis used. Overall, these findings underscore the reliability and concordance of the automated RT-PCR test in the detection of *Bacillus cereus* in donor human milk samples compared to traditional culture-based methods.

### 3.7. Routine Performance of BC Test: Detection of Bacillus cereus in Pre-Pasteurization Donor Human Milk

**Figure 2:**
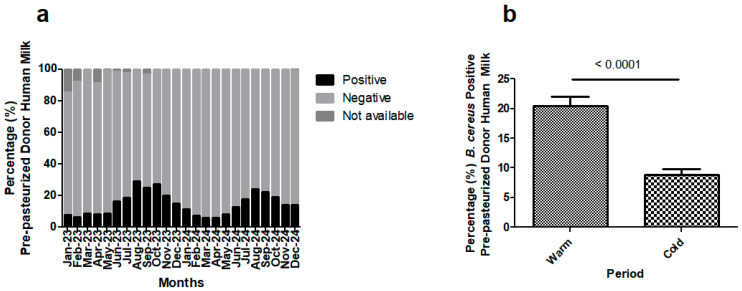
(**a**) Presence of *Bacillus cereus* in pre-pasteurized donor human milk samples, as determined by BC test. Percentage of positive and negative samples. (**b**) Presence of *Bacillus cereus* expressed as percentage of positive pre-pasteurized donor human milk samples, determined by BC test, in warm (June to November) and cold months (December to May). *p* < 0.0001.

From January 2023 until December 2024 (both included), 3439 pre-pasteurized DHM samples were tested to detect *Bacillus cereus* with the newly developed BC test in the cobas^®^ 6800 system. *Bacillus cereus* was detected in 14.2% of DHM samples, with monthly rates ranging from 6 to 29%, as shown in Figure 2. There was a statistically significant (*p* < 0.0001) higher incidence of *Bacillus cereus* in warmer months (June to November) as compared to cold months (December to May). The detection rate, as depicted in Figure 2, was 20.5% (95%CI: 17.24–23.75) in warm months versus 8.9% (95%CI: 7.0–10.7).

## 4. Discussion

In the absence of specific legislation, many HMB operate according to guidelines issued by scientific associations, and thus apply different standards. In addition, as shown in a survey among HMBs in Europe [15], compliance rates with published EMBA and EDQM guidelines are low. For instance, only 33% of HMBs test every sample of pre-pasteurized DHM and only 56% always perform post-pasteurization microbiological tests [15]. This wide variability raises safety concerns. However, the same survey shows that 94% of HMBs in Europe pasteurize DHM. Holder pasteurization eliminates all pathogens that can be found in milk except spores and heat-resistant toxins [3].

*Bacillus cereus* is a significant pathogen due to its ability to cause foodborne diseases; it also produces spores that are resistant to standard Holder pasteurization processes [3,8,9,10]. Its presence in donor human milk (DHM) is of particular concern since it can cause severe infections in premature or ill babies, who have an increased susceptibility to serious infections due to their immature immune systems and underdeveloped gastrointestinal tracts [4,5]. In vulnerable infants, *Bacillus cereus* may cause life-threatening infections, including septicemia, meningitis, respiratory tract infection, hepatitis, endocarditis, endophthalmitis, encephalitis and necrotizing enterocolitis [4,8,9,10]. *Bacillus cereus* can contaminate DHM at all stages, from its collect point to storage and delivery. Although the source of contamination is rarely identified, *Bacillus cereus* in preterm infants should not be overlooked [10]. *Bacillus cereus* produces a diarrhoeagenic toxin that is inactivated by heating and an emetic toxin that resists heat treatment at 126 °C for 90 min [3]. Thus, if the toxin was already present in milk, pasteurization would not eliminate it.

Therefore, fast, accurate and reliable methods to detect *Bacillus cereus* in pre-pasteurization DHM are needed. In recent years, fully automated platforms that perform nucleic acid extraction and RT-PCR with high-throughput capacity, short turnaround time and ease of use have become available. However, there is no commercial kit for the detection of *Bacillus cereus* in automated platforms.

One of such platforms is the cobas^®^ 6800 system [29], which also offers the flexibility to implement self-developed assays. Here we describe the optimization and validation of a RT-PCR assay to be used with the cobas omni Utility Channel in the analyzer cobas^®^ 6800 system (Roche) for the detection of *Bacillus cereus* group in DHM samples. We adapted the assay described by J.F. Martínez-Blanch et al. [25], which had been tested on food products such as liquid egg, cereals or infant formula, but not on human milk. A critical aspect of this study was to determine the optimal extraction volume for milk samples in the cobas^®^ 6800 system. Milk is a complex matrix that can produce interferences in PCR [35] and therefore several alternatives were evaluated. Our results indicated that a small extraction volume (150 µL) after centrifugation was most effective in reducing interference from the milk matrix, while maintaining the efficiency of the RT-PCR process. This protocol minimized the handling of samples, since it only involved a centrifugation step before introducing the samples in the cobas^®^ 6800 system. In contrast, centrifugation and 10-fold dilution were necessary in other studies dealing with human milk [35].

After conducting a risk assessment to identify the parameters that could impact the relevant performance characteristics of the assay, a validation study was designed according to established guidelines [30,31,33].

We assessed the inclusivity and exclusivity using both commercial strains and a comprehensive panel of real 40 DHM samples that had been characterized by culture, 20 positive for *Bacillus cereus* group and 20 negative. Our results demonstrated that the assay could reliably detect *Bacillus cereus* group without cross-reactivity, even when multiple microorganisms were present in the sample at high concentrations. The primers used in our test target the phospholipase gene *plc*, which is present in all the members of the *Bacillus cereus* group [36], and thus increase the inclusivity of the test. This high sensitivity and specificity is essential to reliably detect *Bacillus cereus* especially in DHM, since this matrix is known to harbor a complex microbiota [22,23].

The BC test in cobas^®^ 6800 system exhibited excellent repeatability (intra-assay CV ≤ 1.53%) and intermediate precision over an extended period of time (inter-assay CV ≤ 2.31%). Qualitative results for positive and negative controls remained consistent across different days. Standard deviations were always ≤0.61 Cts. These results are comparable to those obtained for CE-IVD tests performed on automated platforms [32], as well as for other laboratory developed tests [37]. Furthermore, the internal control consistently met required standards in all tests, certifying the lack of interference of milk samples with system’s accuracy. No cross-contamination between negative and positive controls was detected. Therefore, the assay was deemed to be robust.

Establishing the linearity and detection limit of the BC test in a cobas^®^ 6800 system was a key validation aspect. The system demonstrated a strong linear correlation (R^2^ = 0.99) between RT-PCR Ct values and viable bacterial concentrations, in a dynamic range from 10^0^ to 10^7^ CFU/mL in a milk matrix. This range was selected because it agrees with the recommended range (10^0^–10^6^ CFU/mL) set forth by the European Pharmacopeia [33] to validate alternative methods for control of microbiological quality. In our experience, it would allow the detection of typically found levels of contamination in *Bacillus cereus* in milk and is similar to that reported in other molecular assays used to detect *Bacillus cereus* [38].

The limit of detection was verified to be 1 CFU/mL of *Bacillus cereus* in direct DHM samples. This limit of detection is lower than 10^2^ CFU/mL, previously reported for PCR assays in milk with primers targeting *hbl* [26] or *cesB* [39,40], *cytK* and *nheA* [41]. To achieve limits of detection in the range of 1–10 CFU/mL, enrichment by 5–6 h of culture was required in other studies [40,41,42]. A limit of detection of 1 CFU/mL is comparable to the limits achieved by BACARA^®^ culture methods (1 CFU/mL), proving that BC test is effective for detecting low bacterial concentrations in DHM samples, without previous enrichment or extensive pre-treatment.

BACARA^®^ plates are an efficient method to identify and enumerate *Bacillus cereus* group from food matrixes, even in the presence of background flora [24], and are more sensitive and specific than conventionally used media such as mannitol-yolk polymyxin [43,44]. Therefore, BACARA^®^ plate is considered the gold standard for *Bacillus cereus* enumeration. We compared the BC test with the BACARA^®^ plate culture method, revealing robust sensitivity (87.4%) and specificity (93.5%) for the RT-PCR assay. Discrepant samples had a very low concentration of *Bacillus cereus* (1–10 CFU/mL) which probably accounts for the non-repeatability of the results, irrespective of the method of analysis used. Possible causes of the discrepancies include sampling variability and sample integrity. Moreover, RT-PCR’s ability to detect genetic material from non-viable microorganisms may differ from the culture method’s reliance on viable organism for detection. Biological factors such as microbial growth rates, cell viability, and genetic variability can also contribute to discrepancies between the two methods. To avoid detecting non-viable organisms, several authors have developed RT-PCR assays coupled with propidium monoazide, with promising results [26,38,40,41]. Notwithstanding these challenges, Cohen’s Kappa coefficient (0.71) and overall concordance (93.5%) demonstrate substantial agreement [34] between qPCR and culture methods, confirming the BC test as a promising alternative to conventional microbiological techniques for the detection of *Bacillus cereus* in milk.

It is important to highlight that conventional culture-based techniques are laborious and require at least 24–48 h to obtain results. On the other hand, RT-PCR on the fully automated cobas^®^ 6800 system provides results in 2.5 h [45]. Moreover, automated systems reduce human error. Other laboratory-developed assays have also been successfully evaluated on the cobas^®^ 6800 system for bacteria [46] and virus [37] detection. It should be noted that, although our focus was on donor human milk, other food matrices could benefit from this test.

In summary, all parameters recommended by stringent guidelines of method validation [30,31] were evaluated and all fulfilled the acceptance criteria established beforehand in our valuation protocol considering the risk analysis and the intended purpose of the measurement.

This newly developed BC test was used to systematically test all milk donations. In a 24-month period, *Bacillus cereus* was detected in 14.2% of pre-pasteurized DHM samples, with monthly rates ranging from 6 to 29%. We observed a significantly higher presence of *Bacillus cereus* in warmer months, which agrees with previous findings [20]. Although, given the substantial differences in microbiological testing among human milk banks [14,15,18], comparison is difficult, high prevalence of *Bacillus cereus* in DHM has also been described in other studies [20,22,23,47,48,49,50].

Since *Bacillus cereus* resists pasteurization, its presence at any concentration in pre-pasteurization samples is a criterion in BST for rejecting the milk donation before pasteurizing it, to avoid processing contaminated milk and wasting resources. The BC test allows rapid detection of *Bacillus cereus* in less than 3 h and can be decisive in the workflow of milk donation processing.

To the best of our knowledge, this is the first time that a fully automated RT-PCR method to detect *Bacillus cereus* has been developed, implemented, evaluated and routinely used to test pre-pasteurized donor human milk. In addition, in anticipation of future regulatory requirements, this validation has followed stringent guidelines [30,31] that apply to analytical procedures used for release testing of commercial drug substances and products. Although not currently a drug product, recent European regulation [16] that will be applicable from 7 August 2027 has included donor human milk as a Substance of Human Origin and it will have to be controlled following EDQM guidelines, as is the case with blood, cells and tissues. The importance of our contribution is enhanced by the fact that no commercial tests are yet available for the same purpose. Moreover, this test could also be applied to other food matrices.

Our results highlight the importance of using fast, reliable and sensitive detection methods for *Bacillus cereus* in donor human milk, so that contaminated milk donations can be rejected at an early stage before pasteurization takes place, in order to optimize resources, improve safety and minimize health risks to newborn recipients.

The test we have developed and validated could be useful across different milk banks because *Bacillus cereus* is a frequent cause of rejection worldwide [20,22,23,47,48,49,50]. Even though there is currently a lack of global consensus regarding regulation of DHM [14] and therefore there is a large variation in the microbiological quality control of DHM among HMBs [14,15], practices will predictably converge in the future to a more regulated and safe scenario. In this context, fast, automated and easily implemented tests such as the one we here describe may be useful to detect *Bacillus cereus*, a frequently found potential pathogen. Although automated molecular tests might seem more expensive than culture methods, they are in fact easier to implement, even in remote or underdeveloped regions, as they require less experienced personnel [51].

There are, however, limitations to our study such as the existence of discordant results between culture and PCR that could not be resolved. Although culture is still the gold standard, molecular methods can be even more sensitive. Positive samples by PCR and negative by culture could imply very low levels of contamination by *Bacillus cereus* in pre-pasteurized DHM and might lead to excessive discard rates. It remains to be determined if any positive PCR value in pre-pasteurized DHM requires rejection or if DHM batches with low PCR values could undergo pasteurization to decide afterwards if they are acceptable with data from post-pasteurization microbiological controls. We are currently working in this research direction.

## 5. Conclusions

Donor human milk, while essential for infant nutrition, can harbor microbial contaminants that pose a serious risk to vulnerable premature and ill infants due to their immature immune systems. Holder pasteurization is a widely used treatment to mitigate such microbial contamination. However, *Bacillus cereus* is a pathogen frequently found in milk and able to form spores that can survive adverse conditions, including pasteurization processes. Therefore, it is important to detect *Bacillus cereus* during regular pre-pasteurization microbiological testing, so that affected milk donations can be rejected at an early stage before pasteurization.

Here we describe the optimization and validation of a new RT-PCR assay to detect *Bacillus cereus* in donor milk specimens on a fully automated high-throughput platform (cobas^®^ 6800 system). The assay showed excellent inclusivity, specificity, repeatability and linearity, as well as substantial agreement with culture on BACARA^®^ plates. A limit of detection of 1 CFU/mL in donor milk samples was verified. Results were obtained in less than 3 h and could therefore be used to decide if milk donations were suitable for pasteurization or not. The use of this assay in our human milk bank decreased the turnaround time and improved efficiency.

## Figures and Tables

**Table 1:** *Bacillus cereus* (BC) and internal control (IC) results (mean of three replicates) expressed in cycle threshold (Ct) using the cobas^®^ 6800 system. Samples are negative controls (N) or positive controls (P) of BC in donor human milk. ND: no amplification.

N	Extraction Volume	Matrix	Sample	BC Ct	IC Ct	Interpretation
1	850 µL	Unprocessed milk	P	35.20	39.09	Valid
			N	ND	ND	Invalid
2	350 µL	Centrifuged milk (550 µL)	P	34.58	ND	Invalid
			N	ND	37.23	Valid
3	350 µL	Centrifuged milk (400 µL + 150 µL H_2_O)	P	31.98	37.69	Valid
			N	ND	37.19	Valid
4	150 µL	Centrifuged milk (400 µL)	P	28.28	35.75	Valid
			N	ND	35.19	Valid
5	150 µL	Centrifuged milk (250 µL + 150 µL H_2_O)	P	27.45	36.38	Valid
			N	ND	35.39	Valid

**Table 2:** Precision. BC Ct: *Bacillus cereus* cycle threshold. SD: standard deviation. CV: coefficient of variation (%).

Variability	Sample	Replicate	BC Ct	Mean Ct	SD	CV
Intra-run	High positive	1	18.92	18.93	0.29	1.53
		2	18.64			
		3	19.22			
Intra-run	Low positive	1	29.21	29.45	0.25	0.85
		2	29.43			
		3	29.71			
Inter-run	Positive	1	27.30	26.58	0.61	2.31
		2	26.30			
		3	27.73			
		4	27.90			
		5	26.10			
		6	26.80			
		7	26.01			
		8	26,00			
		9	26.43			
		10	26.30			
		11	26.11			
		12	26.25			
		13	26.30			
		14	26.32			
		15	26.92			

**Table 3:** Results of a comparative analysis of the BC test versus the BACARA^®^ plate method for identifying *Bacillus cereus* in donor human milk.

	BACARA^®^ Positive	BACARA^®^ Negative	Total
RT-PCR Positive	210	138	408
RT-PCR Negative	39	1987	2026
Total	309	2125	2434

## Data Availability

The original contributions presented in this study are included in the article/Supplementary Material. Further inquiries can be directed to the corresponding authors.

## Supplementary Materials

The following supporting information can be downloaded at: https://www.mdpi.com/article/10.3390/microorganisms13071640/s1, Table S1: Microbial composition of the Donor Human Milk Samples used to assess inclusivity and specificity.

## Abbreviations

The following abbreviations are used in this manuscript:

RT-PCR	real-time polymerase chain reaction
DHM	donor human milk
Ct	cycle threshold
HMB	human milk bank
HP	Holder pasteurization
BC test	RT-PCR specific for *Bacillus cereus* using the cobas^®^ 6800 system
