# Supplementary material for: Validation of a Real-Time PCR Assay for Fully Automated Detection of Bacillus cereus in Donor Human Milk
Source: Microorganisms. 2025 Jul 11;13(7):1640. doi: 10.3390/microorganisms13071640 (PMC12299169; doi:10.3390/microorganisms13071640)
Supplement: Supplementary file 1 [file microorganisms-13-01640-s001.zip › microorganisms-3713272-supplementary.pdf]

microorganisms

Supplementary Table S1. Sensitivity and Specificity. Microbiological characterization by quantitative culture of the Donor Human Milk specimens used to test sensitivity and specificity and results of RT-PCR for *Bacillus cereus* (BC test). Microbial count expressed in 10^3 Colony Forming Units (CFU) per mL. NA: No Amplification (Ct>45).

N	Microorganism	10^3 CFU/mL	BC Test
1	*Candida parapsilosis*	260	NA
	Stenotrophomonas maltophilia	290	
	Klebsiella oxytoca	190	
	Enterococcus spp	70	
2	*Serratia marcescens*	488	NA
	Staphylococcus epidermidis	30	
	Pseudomonas aeruginosa	20	
3	*Serratia marcescens*	320	NA
	Staphylococcus epidermidis	10	
	Stenotrophomonas maltophilia	230	
	Rhizobium radiobacter	180	
	Acinetobacter junii	10	
4	*Pseudomonas aeruginosa*	160	NA
	Serratia marcescens	400	
	Enterococcus faecalis	40	
5	*Enterobacter cloacae complex*	300	NA
	Acinetobacter ursingii	550	
	Chryseobacterium arthrosphaerae	70	
6	*Klebsiella oxytoca*	30	NA
	Serratia marcescens	330	
	Agrobacterium radiobacter	70	
	Micrococcus luteus	50	
	Acinetobacter ursingii	50	
7	*Micrococcus luteus*	3670	NA
	Pseudomonas aeruginosa	100	
	Chryseobacterium indologenes	20	
8	*Stenotrophomonas maltophilia*	1920	NA
	Agrobacterium radiobacter	20	
	Candida parapsilosis	10	
	Sphingobium yanoikuyae	30	
9	*Micrococcus luteus*	390	NA
	Staphylococcus epidermidis	70	
10	*Micrococcus luteus*	210	NA
	Brachybacterium paraconglomeratum	730	
	Pseudomonas plecoglossicida	110	
	Pseudomonas monteilii	10	
11	*Pseudomonas oryzihabitans*	12,7	NA
	Pantoea anthophila	0,8	
	Staphylococcus epidermidis	0,5	

	Micrococcus luteus	*3160*	
12	*Micrococcus luteus*	*460*	NA
	Enterobacter bugandensis	*10*	
	Chryseobacterium scophthalmum	*20*	
13	*Escherichia hermannii*	*10*	NA
	Klebsiella oxytoca	*150*	
	Enterococcus faecalis	*240*	
	Morganella morganii	*40*	
	Pseudomonas alcaligenes	*10*	
	Candida parapsilosis	*70*	
14	*Acinetobacter nosocomialis*	*520*	NA
	Enterobacter cloacae	*10*	
	Stenotrophomonas maltophilia	*30*	
	Escherichia coli	*10*	
15	*Klebsiella pneumoniae complex*	*124*	NA
	Acinetobacter pittii	*200*	
16	*Serratia nematodiphila*	*290*	NA
	Acinetobacter beijerinckii	*10*	
	Stenotrophomonas maltophilia	*50*	
	Serratia marcescens	*40*	
	Enterococcus faecalis	*130*	
	Staphylococcus epidermidis	*30*	
	Agrobacterium radiobacter	*20*	
	Pseudomonas protegens	*120*	
17	*Pseudomonas aeruginosa*	*320*	NA
	Staphylococcus aureus	*30*	
	Delftia acidovorans	*20*	
	Acinetobacter beijerinckii	*10*	
	Agrobacterium radiobacter	*50*	
	Pseudomonas sp	*10*	
	Klebsiella oxytoca	*20*	
18	*Agrobacterium radiobacter*	*290*	NA
	Chryseobacterium arthrosphaerae	*190*	
	Sphingobium yanoikuyae	*10*	
	Enterobacter cloacae complex	*40*	
	Rhodotorula mucilaginosa	*10*	
	Brevundimonas aurantiaca	*140*	
19	*Klebsiella pneumoniae*	*10*	NA
	Staphylococcus epidermidis	*630*	
20	*Pseudomonas aeruginosa*	*300*	NA
	Serratia liquefaciens	*930*	
	Stenotrophomonas maltophilia	*10*	
	Pseudomonas synxantha	*100*	
21	***Bacillus cereus***	*0,3*	Positive
	Staphylococcus hominis	*0,2*	
22	*Enterobacter hormaechei*	*18*	Positive
	Acinetobacter pittii	*12*	
	Bacillus cereus	*6*	
	Acinetobacter johnsonii	*7*	

	Rhizobium radiobacter	*1*	
	Streptococcus pseudopneumonia	*2*	
23	*Flavobacterium lindanitoleran*	*19*	Positive
	Sphingobacterium multivorum	*3*	
	Rhizobium radiobacter	*27*	
	Enterobacter hormaechei	*2*	
	Bacillus cereus	*1*	
	Acinetobacter ursingii	*26*	
24	***Bacillus cereus***	*11*	Positive
	Pseudomonas sp	*390*	
	Stenotrophomonas maltophilia	*260*	
25	*Enterobacter bugandensis*	*100*	Positive
	Klebsiella oxytoca	*830*	
	Acinetobacter ursingii	*520*	
	Bacillus cereus	*1*	
26	*Pseudomonas monteilii*	*40*	Positive
	Enterobacter cloacae	*880*	
	Acinetobacter beijerinckii	*20*	
	Pseudomonas putida	*1500*	
	Bacillus cereus	*0,3*	
27	*Pseudomonas aeruginosa*	*100*	Positive
	Stenotrophomonas maltophilia	*930*	
	Enterobacter cloacae complex	*510*	
	Bacillus cereus	*0,1*	
28	***Bacillus cereus***	*0,03*	Positive
	Aerococcus viridans	*0,15*	
	Corynebacterium sp.	*0,30*	
	Sphingomonas sp	*0,01*	
29	***Bacillus cereus***	*0,99*	Positive
	Streptococcus oralis	*0,30*	
	Rothia mucilaginosa	*0,50*	
30	***Bacillus cereus***	*0,05*	Positive
	Brevibacillus parabrevis	*0,01*	
31	***Bacillus cereus***	*0,46*	Positive
	Bacillus mycoides	*0,02*	
32	***Bacillus mycoides***	*0,05*	Positive
33	***Bacillus cereus***	*0,01*	Positive
34	***Bacillus cereus***	*1,68*	Positive
35	***Bacillus cereus***	*0,01*	Positive
36	***Bacillus cereus***	*0,30*	Positive
37	***Bacillus cereus***	*0,13*	Positive
38	***Bacillus cereus***	*0,09*	Positive
39	***Bacillus cereus***	*0,01*	Positive
40	***Bacillus cereus***	*0,03*	Positive
